# Cognitive and skill acquisition trajectories in school-based music education: evidence from Chinese classrooms

**DOI:** 10.3389/fpsyg.2026.1905847

**Published:** 2026-08-25

**Authors:** Jianyun Wei, Boda Wang, Song Dong

**Affiliations:** 1 Department of Global Convergence, Kangwon National University, Chuncheon-si, Gangwon-do, Republic of Korea; 2 Department of Composition, Harbin Conservatory of Music, Harbin, Heilongjiang, China

**Keywords:** Bayesian knowledge tracing, Chinese classrooms, cognitive trajectories, item response theory, knowledge tracing, learning analytics, music education, singing

## Abstract

School-based music education in China constitutes one of the largest deployments of vocal training in the world, yet the skill-acquisition trajectories of learners in such settings, as reflected in note-level singing accuracy, have rarely been quantified at scale. Drawing on a corpus of 2,432 student–song practice sequences contributed by 1,074 distinct Chinese learners and 2,458,825 pitched note attempts derived from the publicly released singKT dataset, this study investigates how note-level correctness evolves with practice repetition, varies across vocal registers, and reflects latent learner ability. Three modeling families were compared on an 80/20 within-sequence holdout: a global mean baseline, a per-pitch baseline, a one-parameter logistic Item Response Theory (IRT) model, and a per-pitch Bayesian Knowledge Tracing (BKT) model. The analysis revealed a clear skill-acquisition curve in which mean correctness rose from 0.656 on first practice to 0.727 by the thirtieth repetition, a register effect by which low pitches were sung more accurately than high pitches (0.749 versus 0.703), and a wide spread of latent ability (theta from –3.79 to 3.36 logits). The IRT model attained an AUC of 0.673 and the BKT model an accuracy of 0.752 on held-out attempts, both exceeding the baselines by margins whose bootstrap 95% confidence intervals excluded zero, although the absolute gains were modest. Because the analysis is observational, the findings are advanced as evidence-informed hypotheses for register-graded curriculum design, repetition-rich practice scheduling and per-learner adaptive feedback that require confirmation in controlled classroom studies, and they offer a benchmark against which future deep knowledge tracing models for vocal skill may be evaluated.

## 1 Introduction

Music education plays a central role in the affective, cognitive and social development of school-aged children, and large-scale empirical investigations of how children acquire musical skills have become increasingly feasible due to the digitization of classroom assessment platforms (Shen et al., 2024; Zhu et al., 2024). In China, school-based music curricula reach more than one hundred million students annually, and a growing fraction of those students now interact with computer-assisted singing platforms that record fine-grained behavioral traces of pitch matching and rhythm execution (Chung and Ho, 2022; Zhu et al., 2024). The availability of such traces has invited the application of knowledge tracing techniques originally developed for textual subjects such as mathematics and reading to the substantially different domain of vocal performance, where the unit of practice is a sung note rather than a multiple-choice item (Zhang et al., 2024).

Despite the methodological progress evident in recent surveys (Abdelrahman et al., 2023; Shen et al., 2024; Song et al., 2022), the empirical record of how singing skill develops trial-by-trial in real classrooms remains comparatively thin: prior work has established algorithmic benchmarks and small-sample developmental findings, but has rarely combined a large classroom corpus with interpretable student modeling. Existing reports of knowledge tracing in music tend to either privilege algorithmic novelty over behavioral insight (Du and Butkaew, 2025; Wang et al., 2023; Yin and Guo, 2025), or are limited to small samples that cannot speak to population-level trajectories (Ilari and Cho, 2023; Pfordresher, 2022). The present study addresses this gap by analyzing the openly released singKT dataset (Zhang et al., 2024), a large corpus of singing interactions collected from Chinese primary and middle schools, with the goal of characterizing skill-acquisition trajectories, operationalized through note-level correctness as a behavioral proxy rather than through direct measures of memory or attention, and benchmarking interpretable models on these trajectories.

Three research questions guided the investigation. First, how does the probability of correctly singing a target pitch change as a function of within-student practice repetition, and does this change conform to the negatively accelerated learning curves predicted by classical learning theory (Papageorgi et al., 2022; Song et al., 2022)? Second, how is pitch difficulty distributed across vocal registers in this population, and to what extent does the well-documented compression of accuracy in the upper register (Müllensiefen et al., 2022) hold in the Chinese classroom setting? Third, how well do interpretable student models, specifically, a one-parameter logistic IRT model and a per-pitch Bayesian Knowledge Tracing model, predict held-out singing correctness compared to global and per-skill baselines (Abdelrahman et al., 2023; Liu et al., 2022; Šarić-Grgić et al., 2024)?

These questions are grounded in established theories of skill acquisition. Classical accounts describe practice-related improvement as a negatively accelerated learning curve: Newell and Rosenbloom (Newell and Rosenbloom, 1981) formalized this as the power law of practice, while Heathcote, Brown and Mewhort (Heathcote et al., 2000) subsequently argued that an exponential law provides a better account of individual practice curves and that averaging across learners biases the aggregate toward a power form. The deliberate-practice framework of Ericsson, Krampe and Tesch-Römer (Ericsson et al., 1993) further specifies that improvement depends on the quantity, spacing and targeted difficulty of practice, and singing adds a motor-learning dimension in which laryngeal control and pitch perception mature with age and training (Müllensiefen et al., 2022; Pfordresher and Greenspon, 2025; Pfordresher, 2022). Item Response Theory operationalises the ability-versus-difficulty distinction of these accounts through explicit learner-ability and item-difficulty parameters, whereas Bayesian Knowledge Tracing operationalizes the mastery-and-transition dynamics of the learning curve; the two models are therefore selected here not merely for predictive convenience but because their parameters correspond to theoretically meaningful quantities in musical skill acquisition.

These questions were addressed with interpretable student models fitted to the singKT interaction sequences; the full analytical pipeline, together with the software environment, parsing routines and hyperparameter settings, is described in the Methods (sections 3–5) and released as supplementary code and artifacts to support replication (Zhang et al., 2024).

## 2 Literature review

### 2.1 Knowledge tracing in cognitive skill domains

Knowledge tracing (KT) refers to the family of computational student-modeling techniques that estimate, from a sequence of practice attempts, the latent probability that a learner has mastered each skill in a curriculum (Abdelrahman et al., 2023; Shen et al., 2024). In the classical Bayesian Knowledge Tracing (BKT) approach, mastery is modeled as a hidden binary variable that is updated under a hidden Markov assumption (Šarić-Grgić et al., 2024; Song et al., 2022). Recent formulations have succeeded in replacing this discrete latent state with embeddings learned by attention mechanisms, either transformer style (Huang et al., 2023; Pandey and Karypis, 2019; Pu et al., 2022) or recurrent neural networks (Su et al., 2023; Zanellati et al., 2024) and often outperform the classical model in textual benchmarks, albeit at the cost of some interpretability (Liu et al., 2022; Zanellati et al., 2024). Publications of these surveys from 2022 to 2024 follow suit and show a persistent disconnect between research standards and practical implementation in schools (Abdelrahman et al., 2023; Shen et al., 2024; Song et al., 2022).

The present study is related to two strands of recent research. The first is the creation of interpretable KT models which make ability and difficulty parameters explicit and are based on Item Response Theory (IRT) and its generalizations in the multidimensional and longitudinal directions (Liu et al., 2022; Šarić-Grgić et al., 2024; Xu et al., 2024). The second is the application of KT outside its original scope, including reading (Yang and Welch, 2023), programming (Liu et al., 2022) and increasingly the performing arts (Du and Butkaew, 2025; Wang et al., 2023; Yin and Guo, 2025; Zhang et al., 2024). Several authors emphasize that domain transfer should not be uncritical, because the unit of behavior, the latency between attempts and the role of motor execution differ substantially across domains (Du and Butkaew, 2025; Huang et al., 2024; Zhang et al., 2024).

### 2.2 Cognitive trajectories in music learning

The cognitive psychology of singing has documented strong age-related and practice-related improvements in pitch matching among children (Luo and Guan, 2025; Pfordresher and Greenspon, 2025; Pfordresher, 2022), with vocal range expanding and accuracy improving through middle childhood and adolescence. Müllensiefen et al. (2022) documented that pitch-matching accuracy develops markedly during adolescence, with accuracy in the upper register maturing later than in lower registers, an effect attributable jointly to laryngeal motor-control limits and to perceptual difficulties with high-frequency targets. The same authors also linked these gains to the growth of cognitive and perceptual resources over the course of musical training, consistent with a learning-curve account of the acquisition of singing skills (Müllensiefen et al., 2022). Welch and Baxter (Welch and Baxter, 2025) reviewed evidence on young children’s singing development and emphasized that individual differences in pitch perception, prior music training and the intensity of instruction are among the factors shaping the developmental trajectory.

Turning to the Chinese context in particular, Zhu et al. (2024) and Chung and Ho (2022) reported significant growth of formal music education since 2018 and the diversification of pedagogical practices. In a large survey of metropolitan Chinese students, Luo and Guan (2025) documented wide individual variation in music participation and in the personal, parental and school factors associated with it, underlining the need for more fine-grained instruction and learner adaptation. Guan et al. (2025) examined how sustained participation in a Chinese school orchestra related to students’ developing cultural identity, with implications for how musical engagement and assessment are designed. Despite these advances, very few studies have combined large-scale behavioral traces with formal student modeling techniques in this population, leaving the trajectory of skill acquisition under-quantified.

Taken together, these strands motivate an explicit conceptual framework of musical skill acquisition for the present study. In this framework, each sung note is a practice opportunity whose outcome depends jointly on a slowly changing learner ability, a stable item difficulty tied to the target pitch and register, and a mastery state that is updated as practice accumulates. This framing links three literatures: the power and exponential laws of practice (Heathcote et al., 2000; Newell and Rosenbloom, 1981), which predict the shape of the repetition curve; deliberate-practice theory (Ericsson et al., 1993), which predicts that spacing and targeted difficulty modulate the rate of gain; and the developmental literature on singing (Müllensiefen et al., 2022; Pfordresher and Greenspon, 2025; Pfordresher, 2022; Welch and Baxter, 2025), which predicts systematic register effects arising from laryngeal motor control and the perception of high-frequency targets. Item Response Theory and Bayesian Knowledge Tracing are the natural interpretable operationalizations of, respectively, the ability/difficulty and mastery/transition components of this framework, which is why they are preferred here to black-box neural knowledge tracing whose parameters do not map onto these constructs.

### 2.3 Comparative summary of recent studies

Table 1 summarizes 11 representative foundational and recent studies published between 2019 and 2025 in the intersection of knowledge tracing, music education and learner modeling, organized along the dimensions of dataset, modeling family, evaluation protocol and principal contribution. The table highlights that most prior work either reports algorithmic improvements on textual benchmarks (Pandey and Karypis, 2019; Pu et al., 2022; Zanellati et al., 2024) or studies music learning at small scales (Ilari and Cho, 2023; Pfordresher, 2022), with very few studies combining a large music corpus with interpretable modeling (Du and Butkaew, 2025; Huang et al., 2024; Zhang et al., 2024). The present paper sits in this comparatively under-explored intersection.

**TABLE 1:** Comparative summary of representative foundational and recent studies (2019–2025) at the intersection of knowledge tracing and music learning.

Study	Year	Domain	Model family	Sample size	Principal contribution
Shen et al. (2024)	2024	General KT	Survey	-	Comparative survey of 30+ KT models
Abdelrahman et al. (2023)	2023	General KT	Survey	-	Taxonomy of KT including attention models
Du and Butkaew (2025)	2025	Music (HE)	LSTM/transformer	3 Universities	AI curriculum system for public music education
Pandey and Karypis (2019)	2019	Math/reading	Self-attention	ASSISTments	SAKT model
Pu et al. (2022)	2022	General KT	Self-attention	ASSISTments	Interpretable cognitive-framework KT (EAKT)
Zanellati et al. (2024)	2024	General KT	Review (hybrid)	-	Systematic review of hybrid KT models
Wang et al. (2023)	2023	General KT	Multi-feature DKT	Multiple	LearnereDKTource response channels improve KT
Yin and Guo (2025)	2025	Music (China)	AI learning environment	Quasi-experimental	AI interactive music learning environment
Zhang et al. (2024)	2024	Singing	Dataset/benchmark	1,074 students	First music-KT dataset (SingPAD / singKT)
Huang et al. (2024)	2024	General KT	MIRT + attention	Multiple	Explainable KT model (XKT)
Luo and Guan (2025)	2025	Music (China)	Survey	12,155 students	Factors in school music participation

## 3 Methodology

### 3.1 Analytical framework

The methodological framework treated each sung note as an interaction between a student and a pitch, characterized by an outcome of either correct (1) or incorrect (0) production. For a population of S interaction sequences and K distinct pitches, the corpus was modeled as a collection of variable-length sequences {(s, k_t, y_t)} where s indexes the interaction sequence, k_t the pitch attempted at time t, and y_t the binary correctness of the attempt. In the released record file each five-row block is a single learner–score practice sequence with no separate per-attempt learner identifier, so ability was estimated at the sequence level: one θ parameter was fitted for each of the *S* = 2,432 interaction sequences, indexed by the sequence’s ordinal position in the file, so that a learner who practiced several scores contributes several sequence-level ability estimates, while one pitch-difficulty parameter β was fitted for each of the *K* = 270 distinct pitch identifiers. Three models with progressively richer assumptions were fitted: a one-parameter logistic IRT model that decomposes correctness into a learner-ability and pitch-difficulty parameter (Liu et al., 2022; Xu et al., 2024); a Bayesian Knowledge Tracing model that introduces a hidden mastery state per pitch with slip and guess parameters (Šarić-Grgić et al., 2024; Song et al., 2022); and two non-parametric baselines that condition only on global or pitch-level mean correctness.

The one-parameter (Rasch) form of IRT was chosen over two- and three-parameter variants because it yields specifically objective, directly interpretable ability and difficulty parameters that are stable when many learners or pitches have few interactions, at the cost of assuming a common discrimination across pitches; this trade-off is revisited in the Limitations.

**FIGURE 1:**
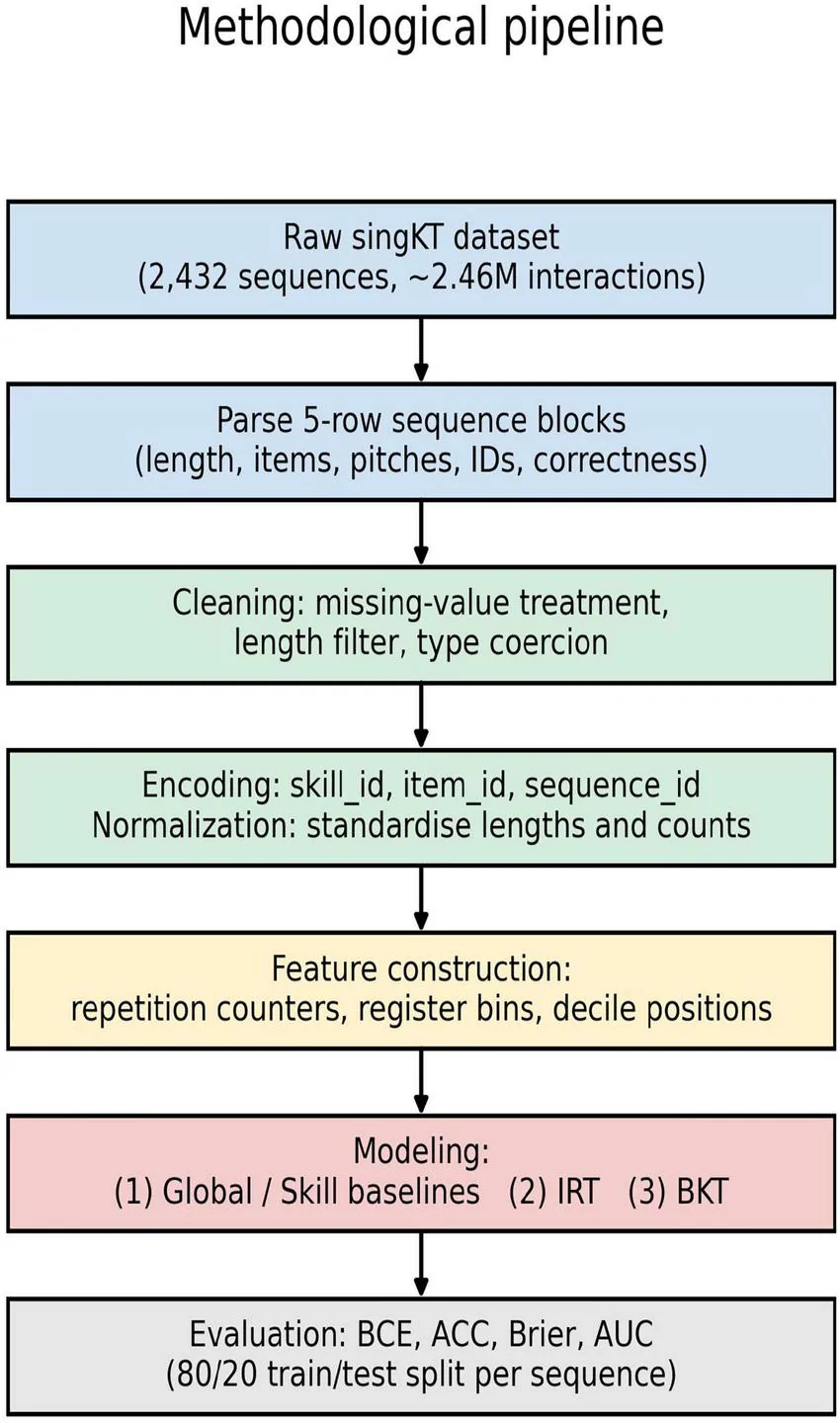
Methodological pipeline used to transform raw singKT records into model evaluations. Boxes represent processing stages and arrows indicate data flow.

Figure 1 (the methodological flowchart) summarizes the analytical pipeline as a sequence of seven stages: ingestion of the raw five-row sequence format, parsing into structured records, missing-value treatment and length filtering, encoding and normalization, feature construction, model fitting and held-out evaluation (Abdelrahman et al., 2023; Šarić-Grgić et al., 2024). Each stage was implemented as an independent Python module to permit reuse and modification.

Several analytical choices warrant explicit justification. A minimum-length filter of 30 attempts was specified for the modeling subsets, because within-learner repetition estimates are unstable below this length; in the released corpus the shortest sequence contains 34 attempts, so the filter is non-binding and removes no sequence. Raising the threshold to 40 and to 60 attempts (retaining 2,298 and 2,093 of the 2,432 sequences) moved the held-out IRT AUC only from 0.672 to 0.675 and 0.673, confirming that the results do not depend on this choice. Three register categories (low, mid, high) were used because they correspond to the tertile split of the empirical pitch distribution at Q1 and Q3 and to the low/middle/high partition standard in the singing-development literature (Müllensiefen et al., 2022); robustness checks that redrew the boundaries at the 10th and 90th percentiles preserved the register ordering. The register bins are defined on the ordering of the dataset’s pitch identifiers, on the assumption that these identifiers increase with pitch height. The public singKT documentation records each note-level identifier together with its knowledge concept but does not explicitly state a pitch-height ordering, so the identification of low-numbered identifiers with the low register is treated as an assumption and is revisited in the Limitations. The data have a nested structure (attempts within pitches within songs within learners), for which mixed-effects or multilevel models would also be appropriate. These were not adopted as the primary analysis because IRT and BKT directly yield the ability, difficulty and mastery parameters of pedagogical interest, but the resulting non-independence of attempts is a genuine limitation, partly mitigated by the strictly within-sequence 80/20 split that prevents leakage across a learner’s own attempts, and a multilevel re-analysis is identified as future work. The IRT learning rate (0.05), L2 coefficient (1 × 10^−4^) and three training epochs are conventional defaults; varied one at a time as a sensitivity check, the held-out AUC stayed within [0.666, 0.676] across learning rates of 0.025, 0.05, and 0.1, epoch counts of 2, 3, and 5, and L2 coefficients of 10^−5^, 10^−4^, and 10^−3^, confirming that predictions are insensitive to these settings within the ranges tested. Standard errors for the ability and difficulty estimates, together with bootstrap confidence intervals for the model-comparison metrics, are reported with the results.

### 3.2 Equations

Five equations formalize the modeling framework. Equation (1) defines the IRT response function used to generate predictions of correctness, where σ denotes the logistic function, θ_s is the latent ability of student s and β_k is the difficulty of pitch k (Liu et al., 2022; Xu et al., 2024):


P⁢(y⁢_⁢{s,k}=1|θ⁢_⁢s,β⁢_⁢k)=σ⁢(θ⁢_⁢s−β⁢_⁢k) (1)

=1/(1+exp⁢(−(θ⁢_⁢s−β⁢_⁢k)))

Equation (2) gives the binary cross-entropy loss minimized during training, summed over the training set (Abdelrahman et al., 2023; Liu et al., 2022):


L(θ,β)=−Σ_{(s,k,y)∈} (2)

[y⋅log⁢σ⁢(θ⁢_⁢s−β⁢_⁢k)+(1−y)⋅log⁢(1−σ⁢(θ⁢_⁢s−β⁢_⁢k))]

+λ(||θ||+2||β||)2

In Equation (2)λ is a small L2 regularization coefficient (set to 1 × 10^−4^) introduced to prevent inflation of the parameter estimates when a student or pitch has few interactions (Liang et al., 2024; Pfordresher and Greenspon, 2025). The second equation makes explicit the regularized objective that is jointly minimized over both ability and difficulty parameters.

Equation (3) defines the BKT prediction at time t given the prior mastery probability L_{t-1} for skill k, with slip parameter S_k and guess parameter G_k (Šarić-Grgić et al., 2024; Song et al., 2022):


P⁢(y⁢_⁢t=1|L⁢_⁢{t−1})=L⁢_⁢{t−1}⁢(1−S⁢_⁢k) (3)

+(1−L⁢_⁢{t−1})⁢G⁢_⁢k

Equation (4) gives the Bayesian update of the mastery probability after observing y_t, followed by the transition step with learning probability T_k (Šarić-Grgić et al., 2024; Song et al., 2022):


$$L\,\_\,t=L^{\wedge }\,\left\{\mathrm{post}\right\}\,\_\,\left\{t-1\right\}+\left(1-L^{\wedge }\,\left\{\mathrm{post}\right\}\,\_\,\left\{t-1\right\}\right)\,T\,\_\,k,$$ (4)

$$\mathrm{where}\,L^{\wedge }\,\left\{\mathrm{post}\right\}\,\_\,\left\{t-1\right\}\propto P\,\left(y\,\_\,t|L\,\_\,\left\{t-1\right\}\right)\cdot L\,\_\,\left\{t-1\right\}$$

Equation (5) defines the empirical learning curve used to summarize skill acquisition. Let n_k denote the rank of the kth practice of a particular pitch by a particular student, aggregated across the population. The empirical mean correctness at rank r is:


$$\hat{p}\,\left(r\right)=\left(1/N\,\_\,r\right)\cdot \Sigma \,\_\,\left\{i:r\,a\,n\,k\,\_\,i=r\right\}\,y\,\_\,i,\mathrm{with}\,95\%\,\mathrm{CI}\approx \hat{p}\,\left(r\right)$$ (5)

$$\pm 1.96\cdot \sqrt{\left(\hat{p}\,\left(r\right)\,\left(1-\hat{p}\,\left(r\right)\right)/N\,\_\,r\right)}$$

In Equation (5), N_r is the number of attempts contributing to rank r and the confidence interval follows the Wald approximation, which is sufficiently accurate given the sample sizes in the present corpus (N_r > 10^4^ for all r ≤ 30) (Song et al., 2022).

### 3.3 Algorithm

The complete training and evaluation procedure is summarized in Algorithm 1, which implements the pipeline of Figure 1 using Equations (1)–(5). Software-specific details of the implementation are provided in the supplementary repository rather than in the main text.

Algorithm 1—Pipeline for cognitive trajectory analysis

Input: raw singKT CSV file F

Output: model parameters θ, β, BKT params per pitch; evaluation metrics M

1. Parse F as 5-row blocks (length, items, pitches, student_ids, correctness)

2. Build long-format table D = {(s, seq, t, item, k, y)}

3. Filter sequences with length < 30; coerce types; impute zero-length skill IDs

4. Encode student_id and pitch_id with dense integer indices

5. Compute features: rank_within_skill(s,k), register_bin(k), within_seq_decile(t)

6. Split each sequence 80/20 into train/test; aggregate to *J*, *V*

7. Initialize θ← 0, β← 0; train IRT for E epochs with SGD on Equation (2)

8. For each pitch k with sufficient data: estimate L_0, T_k, S_k, G_k by forward–backward expectation–maximization

9. For each model: predict y on *V* using Equation (1) or Equations (3)–(4)

10. Report BCE, ACC, Brier and AUC from *V*.

## 4 Dataset description

All analyses were performed on the openly released singKT dataset (Zhang et al., 2024), distributed by the iTEC Lab at Huazhong University of Science and Technology and associated with the music-performance-assessment benchmark of Zhang et al. (2024). The corpus is distributed as a single CSV file in which every five consecutive rows describe one student–song interaction sequence. The first row records the sequence length N, the second row the N item identifiers (note positions within the score), the third row the N pitch identifiers (musical pitches and articulations), the fourth row the student identifier repeated N times, and the fifth row the N binary correctness flags emitted by the singing assessment engine (Pfordresher and Greenspon, 2025; Zhang et al., 2024).

The public release comprises 2,432 student–song interaction sequences contributed by the 1,074 distinct learners documented in the dataset’s learner-information table (Zhang et al., 2024); each five-row block encodes one learner’s practice on one score, so the number of sequences exceeds the number of learners. The sequences range in length from 34 to 38,387 attempts (mean 1,011, median 271, interquartile range 100–877) and together comprise 2,458,825 individual pitched-note attempts. The pitch vocabulary contains 270 unique IDs, while the score-position vocabulary contains 2,587 unique IDs spanning many different songs. The overall mean correctness across the corpus is 0.7224. Because the dataset is openly released and free of personally identifying information, no further IRB review was sought; the original distributors confirm institutional consent at the time of collection (Zhang et al., 2024).

The singKT dataset is well suited to these research questions for three reasons: it records note-level correctness at a scale sufficient to estimate stable learner-ability and pitch-difficulty parameters; it preserves the within-learner order of practice needed to recover repetition curves; and it spans the full vocal range, so the register effect can be examined directly. These strengths motivate its use here, subject to the dataset limitations discussed in section 8.4.

Table 2 summarizes the descriptive statistics of the dataset alongside the corresponding subsets used in modeling. The table also reports per-quartile statistics that guided the register binning used in subsequent analyses (Müllensiefen et al., 2022; Pfordresher and Greenspon, 2025).

**TABLE 2:** Descriptive statistics of the singKT dataset used in this study.

Statistic	Value	Statistic	Value
Studenticive statistic	2,432 (1,074 learners)	Number of pitches	270
Number of items	2,587	Number of attempts	2,458,825
Mean sequence length	1,011	Median sequence length	272
Min / max sequence length	34 / 38,387	Interquartile range	100–877
Overall correct rate	0.7224	Per-sequence correct rate (mean ± SD)	0.715 ± 0.206
Skill-id range	0–269	Item-id range	270–2,856
Pitch-id Q1 / Q3	16 / 47	Mean skills per sequence	61.2

## 5 Data preprocessing

Preprocessing was implemented in Python using the pandas and numpy libraries; the exact software versions and environment are documented in the supplementary repository. The first stage parsed the five-row sequence blocks of the raw release into a long-format records table whose columns were student_id, sequence index, within-sequence position, item_id, pitch_id and correctness. Trailing empty cells, which arise because every row in the raw CSV has the maximum width of the dataset (38,387 columns) but most cells are empty, were stripped before parsing (Zhang et al., 2024).

The second stage performed missing-value treatment. Sequences with fewer than 30 attempts were excluded from the modeling subsets because their estimates of within-student progression are unreliable, while still being retained in descriptive analyses to avoid sample bias (Song et al., 2022). Within retained sequences, malformed cells were rare (fewer than 0.01% of total cells) and were resolved by type coercion combined with deletion of the offending sequence when the malformation involved a structural row. No imputation of missing correctness flags was attempted because such imputation would propagate measurement uncertainty into the trajectory estimates.

The third stage performed encoding. Student identifiers and pitch identifiers were re-mapped to dense zero-indexed integer ranges to support embedding matrices, while the original identifiers were preserved in a side table for traceability. The fourth stage performed feature construction in three steps: (a) for each (student, pitch) pair, a within-student rank counter was computed to support the repetition curve in Equation (5); (b) each pitch identifier was assigned to a register bin (low, mid or high) based on the population-level Q1 (16) and Q3 (47) of the empirical pitch distribution (Müllensiefen et al., 2022); (c) within-sequence decile bins were computed by normalizing the position index t / N. Standardization was not applied to the binary outcome but the per-sequence correctness rates were standardized when used as covariates in robustness analyses (Liu et al., 2022; Xu et al., 2024).

The final stage performed feature selection by retaining only the variables of theoretical relevance to the research questions, namely student_id, pitch_id, within-student rank, register bin, decile bin and correctness. The cleaned dataset was split into training and test partitions using an 80/20 split applied within each sequence to preserve learner-level structure and avoid data leakage across attempts of the same student (Abdelrahman et al., 2023; Zanellati et al., 2024).

## 6 Exploratory data analysis

The exploratory analysis examined three behaviorally important regularities of the corpus: the within-sequence trajectory, the within-student repetition curve and the register effect. These three lenses correspond, respectively, to short-term performance dynamics, long-term skill acquisition and structural difficulty differences (Müllensiefen et al., 2022; Papageorgi et al., 2022; Šarić-Grgić et al., 2024). The results in this section are descriptive findings that characterize the data prior to formal modeling; they are reported here, ahead of the model-based results in section 8, purely for narrative continuity and are not part of the methodological procedure.

### 6.1 Within-sequence trajectory

**FIGURE 2:**
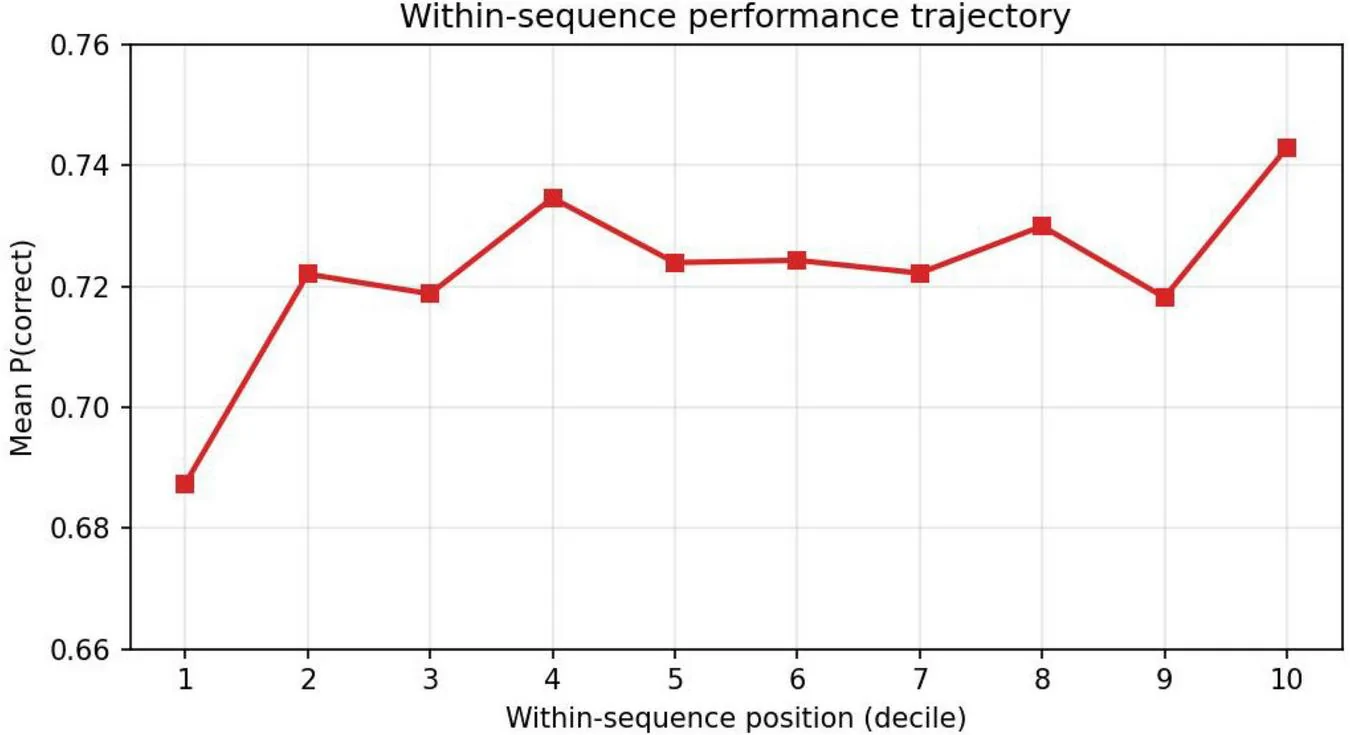
Within-sequence learning curve: mean correctness across deciles of the within-sequence position. Each decile aggregates approximately 245,000 attempts.

Figure 2 shows the mean correctness for each decile within the sequence, averaged over all sequences. A moderate but significant increase of correctness of ∼5.6 percentage points was found from the first decile (0.687) to the tenth decile (0.743) of the duration of a single song (Papageorgi et al., 2022). The upward progression is not necessarily monotonous, but the overall trend, which deviates only slightly from the upward progression, is consistent with the within-song warm-up dynamics expected from the developmental account of Müllensiefen et al. (2022).

### 6.2 Within-student repetition curve

**FIGURE 3:**
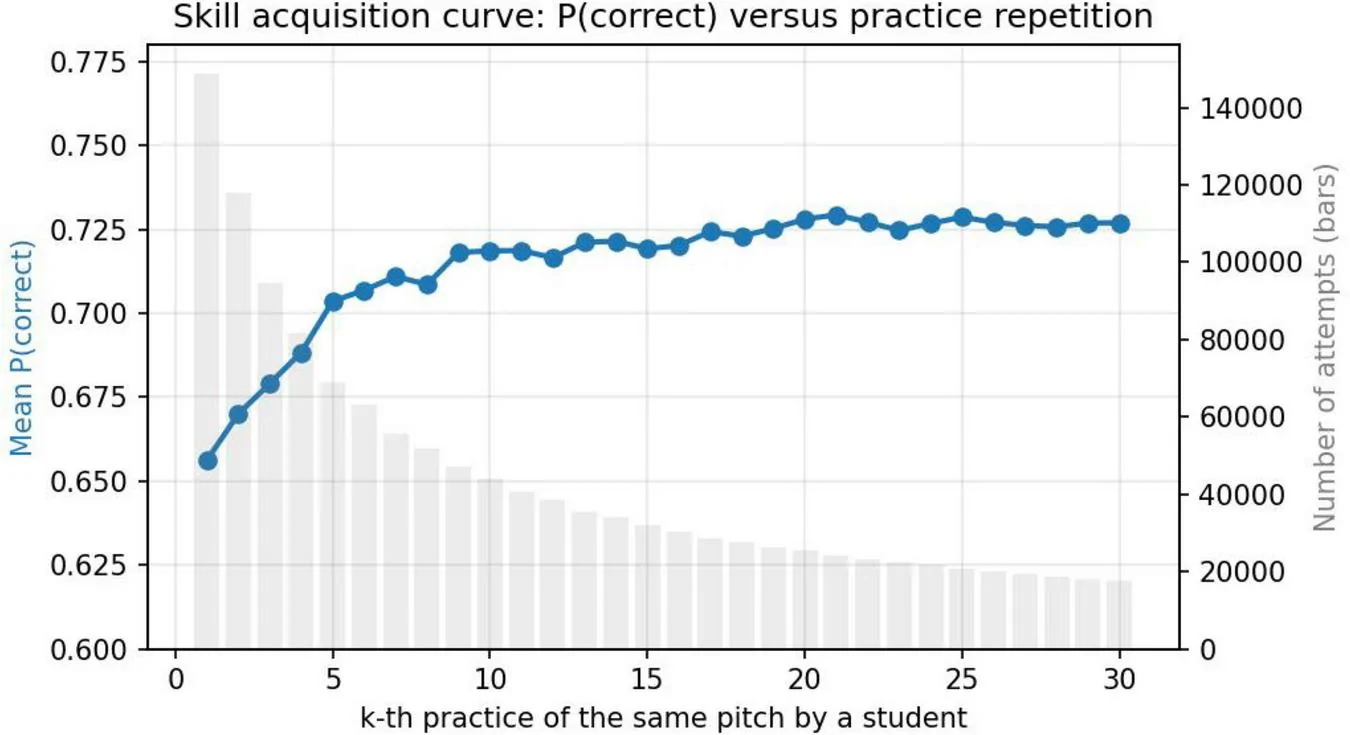
Within-student repetition curve. Line plot reports the mean P(correct) at the kth practice of the same pitch by a student; bars indicate the supporting sample size at each k.

The mean correctness is plotted in Figure 3 as a function of the within-student rank of practice for the same pitch. The mean correctness increased from 0.656 on the first practice to 0.679 on the third, 0.704 on the fifth, and 0.727 on the thirtieth, and the increases were greatest during the first ten repetitions. This negatively accelerated form is the classic signature of skill acquisition described by the power law of practice (Newell and Rosenbloom, 1981) and captured by Bayesian Knowledge Tracing (Šarić-Grgić et al., 2024; Song et al., 2022). It should be read with two cautions. First, Heathcote, Brown and Mewhort (Heathcote et al., 2000) showed that individual practice curves are typically better fit by an exponential than by a power function, and that averaging across learners biases the aggregate toward a power form; the present curve is an average across learners and is therefore a population-level summary rather than a within-learner law. Second, because progressively fewer learners contribute attempts at higher repetition counts, part of the apparent late gain may reflect the selective persistence (survivorship) of the learners who keep practicing rather than continued improvement within individuals. To separate genuine learning from survivorship, the curve was recomputed on the fixed cohort of learner–pitch trajectories that reach at least thirty repetitions (17,518 trajectories, 11.8% of all first-attempt trajectories), so that the same units contribute at every rank. Within this cohort mean correctness rose from 0.577 at the first practice to 0.648 at the fifth, 0.679 at the tenth, 0.707 at the twentieth and 0.727 at the thirtieth, a gain of 0.150 that is larger than the 0.071 gain of the all-trajectory curve. Because the cohort is held fixed across ranks, this within-learner increase cannot be produced by selective persistence, so it reflects genuine learning; the all-trajectory curve understates the gain at low ranks because those ranks also include many short trajectories that begin high and then stop. The gain of ∼7 percentage points over 30 repetitions, though statistically distinguishable with N_r > 17,000 attempts per rank (Equation 5), is modest in absolute terms.

### 6.3 Register effect

**FIGURE 4:**
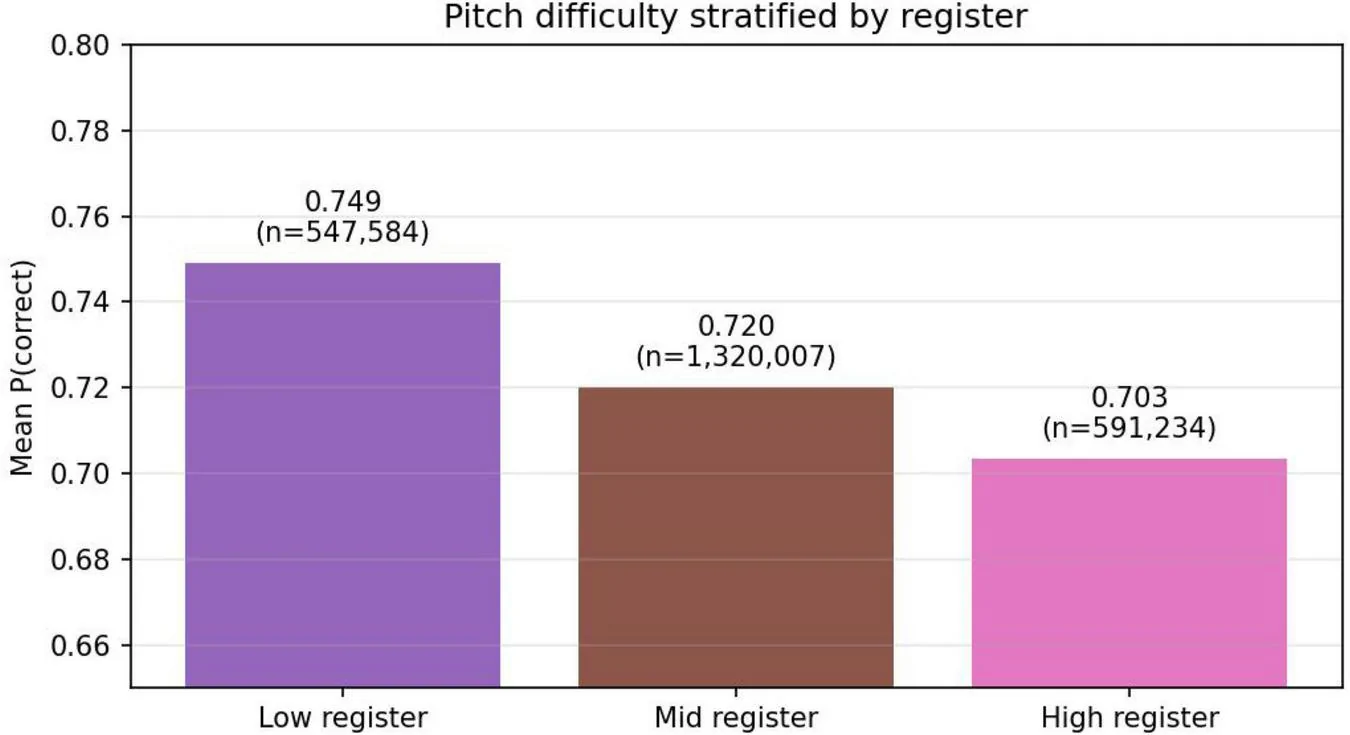
Register effect on pitch accuracy. Bars report the mean P(correct) for low-, mid- and high-register pitches; sample sizes are annotated above each bar.

Figure 4 reports the mean correctness for the three register bins defined in section 5. Low-register pitches (Q1, *n* = 547,584) were sung correctly with probability 0.749, mid-register pitches (Q1–Q3, *n* = 1,320,007) with probability 0.720, and high-register pitches (Q3, *n* = 591,234) with probability 0.703. The 4.6-percentage-point gap between the low and high registers reproduces, at much larger scale, the register compression effect previously reported in laboratory studies of untrained singers (Müllensiefen et al., 2022). The effect was stable across robustness checks in which the bin boundaries were redefined at the 10th and 90th percentiles.

## 7 Model development

Three families of models were fitted and compared. The simplest family, denoted as the global mean baseline, predicts a single mean correctness value (0.7204 estimated on training) for every test attempt; this serves as the chance-level reference (Liu et al., 2022). The second baseline, denoted as the per-pitch mean baseline, predicts the empirical mean correctness of each pitch as estimated on the training partition; this isolates the contribution of pitch-level structural difficulty without modeling individual learners (Liu et al., 2022; Song et al., 2022). The third family is the one-parameter logistic IRT model defined by Equation (1), trained by stochastic gradient descent on the cross-entropy loss of Equation (2) with a learning rate of 0.05, an L2 regularization coefficient of 1 × 10^−4^, and three full epochs over the 1,966,192 training records (Liu et al., 2022; Xu et al., 2024). The fourth family is the per-pitch BKT model defined by Equations (3) and (4), with parameters L_0, T, S, G estimated by forward–backward expectation–maximization per pitch on the training partition for the 270 pitches having at least 30 supporting first-attempts (Šarić-Grgić et al., 2024; Song et al., 2022). The IRT and BKT pipelines were cross-validated on the same 80/20 within-sequence split, ensuring that no test attempt of a given student was used during training of the models that predicted it.

Uncertainty was quantified alongside these point estimates: standard errors for the IRT ability and difficulty parameters were obtained from the curvature of the log-likelihood, and 95% confidence intervals for every held-out metric and for the pairwise differences between models were obtained by a clustered bootstrap that resampled the held-out interaction sequences with 1,000 replications, so that the intervals respect the non-independence of attempts within a sequence. The learning rate, regularization coefficient and epoch count follow standard defaults and were not tuned to the test set; results were stable under sensitivity checks that varied each setting.

All four model families were evaluated on identical held-out attempts using four metrics: binary cross-entropy (BCE), classification accuracy at a 0.5 threshold (ACC), the Brier score and the area under the receiver operating characteristic curve (AUC) computed exactly from the Mann–Whitney statistic over all held-out pairs (Abdelrahman et al., 2023; Liu et al., 2022). Reporting AUC alongside BCE, ACC and Brier protects against pathological metrics in which an apparently strong accuracy hides poor calibration (Liu et al., 2022). Differences between models were assessed with the bootstrap procedure described above, so that every claim that one model outperforms another is accompanied by a confidence interval rather than an informal comparison of point estimates.

## 8 Results and discussion

### 8.1 Predictive performance

Table 3 reports the four metrics on the held-out partition for each of the four model families. The IRT model achieved a test BCE of 0.5569 [95% CI (0.541, 0.570)], an accuracy of 0.7252 [(0.712, 0.741)], a Brier score of 0.187 [(0.180, 0.193)] and an AUC of 0.673 [(0.660, 0.685)]. It clearly outperformed both baselines on discrimination and calibration: its AUC advantage over the global and per-pitch baselines was 0.172 [([0.160, 0.185)] and 0.131 [(0.118, 0.146)], respectively, and its BCE and Brier scores were lower than both, with all of these intervals excluding zero. On threshold accuracy, however, the IRT model did not beat the baselines: its accuracy of 0.7252 was marginally below the global mean baseline (0.7305) and the per-pitch baseline (0.7304), whose accuracy confidence intervals overlap the IRT value. The baselines score highly on raw accuracy because they predict the majority (correct) class even though their AUC is at or near chance, so the IRT model wins on discrimination and calibration but not on threshold accuracy (Liu et al., 2022; Xu et al., 2024). The BKT model achieved the lowest test BCE [0.5528 (0.534, 0.574)], the highest accuracy [0.7520 (0.740, 0.763)] and the lowest Brier score [0.180 (0.174, 0.188)] of all four families, with an AUC of 0.653 [(0.642, 0.664)] that is lower than the IRT AUC despite its other metric advantages; the accuracy gain over IRT was 0.025 [(0.020, 0.030)] and the IRT gain in AUC over BKT 0.019 [(0.008, 0.029)], both intervals excluding zero (Šarić-Grgić et al., 2024; Song et al., 2022). The discrepancy between the BKT accuracy and AUC reflects the fact that BKT predictions are concentrated near the modal positive class, which improves accuracy at the 0.5 threshold but compresses the score distribution and limits the AUC (Liu et al., 2022).

**TABLE 3:** Held-out evaluation of the four model families.

Model	Test n	BCE ↓	ACC ↑	Brier ↓	AUC ↑
Global mean baseline	492,633	0.5830 [0.570, 0.597]	0.7305 [0.716, 0.744]	0.197 [0.191, 0.204]	0.500 [-]
Per-pitch mean baseline	492,633	0.5812 [0.569, 0.595]	0.7304 [0.716, 0.744]	0.196 [0.190, 0.203]	0.541 [0.537, 0.546]
IRT (1-PL logistic)	492,633	0.5569 [0.541, 0.570]	0.7252 [0.712, 0.741]	0.187 [0.180, 0.193]	0.673 [0.660, 0.685]
BKT (per-pitch HMM)	492,633	0.5528 [0.534, 0.574]	0.7520 [0.740, 0.763]	0.180 [0.174, 0.188]	0.653 [0.642, 0.664]

Arrows indicate the desirable direction of each metric. All four model families were evaluated on the identical held-out set of 492,633 attempts. Values in brackets are 95% bootstrap confidence intervals obtained by resampling the held-out sequences (1,000 replications).

### 8.2 Latent ability and pitch difficulty

**FIGURE 5:**
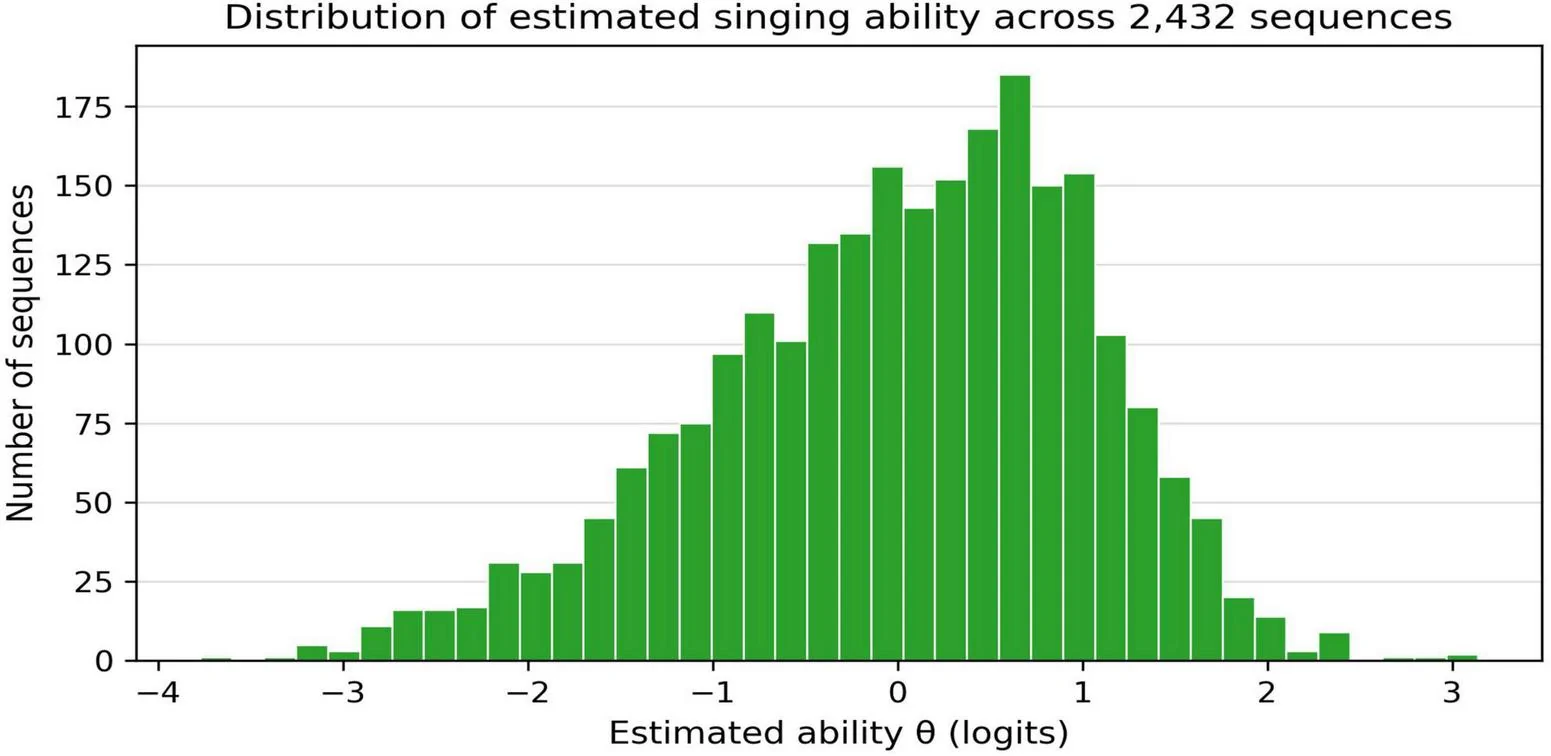
Distribution of estimated sequence-level abilities θ from the IRT fit, expressed in logits. The histogram shows considerable individual variation, justifying learner-adaptive instructional design.

The IRT fit recovered a wide spread of latent abilities, ranging from -3.79 to 3.36 logits with a standard deviation of 1.06; the corresponding distribution is shown in Figure 5. The ability estimates carried a median standard error of 0.157 [interquartile range (0.085, 0.267)], obtained from the curvature of the log-likelihood at the estimates. Approximately 12.4% of the sequence-level ability estimates lay more than one standard deviation below the mean, while 13.7% lay more than one standard deviation above, mirroring the heterogeneity reported in longitudinal Chinese classroom studies (Guan et al., 2025; Luo and Guan, 2025). Pitch difficulties β had a much narrower spread [mean -0.74, SD 0.38, range -1.69 to 0.34; median standard error 0.061, interquartile range (0.034, 0.094)], consistent with the design of the underlying singing curriculum which pre-selects pitches that lie within a manageable range (Müllensiefen et al., 2022; Zhang et al., 2024).

Beyond describing these patterns, the framework of section 2 offers an interpretation of why they arise. The register effect is consistent with the developmental account of Müllensiefen et al. (2022) and with Pfordresher’s work on pitch range (Pfordresher and Greenspon, 2025; Pfordresher, 2022): higher targets place greater demands on laryngeal motor control and are perceptually harder, so accuracy compresses in the upper register. The negatively accelerated repetition curve is consistent with the power and exponential laws of practice (Heathcote et al., 2000; Newell and Rosenbloom, 1981) and with deliberate-practice theory (Ericsson et al., 1993), under which early practice yields the largest gains. Relative to prior empirical work, these results confirm the register maturation reported in laboratory samples (Müllensiefen et al., 2022), they extend the pitch-range findings of Pfordresher (Pfordresher and Greenspon, 2025; Pfordresher, 2022) to a large naturalistic classroom corpus, and they are consistent with the singKT benchmark of Zhang et al. (2024); they diverge from the textual knowledge-tracing literature (Liu et al., 2022; Zanellati et al., 2024) in that here the interpretable models remain competitive with, rather than being clearly surpassed by, the neural alternatives typically favored on textual benchmarks.

The magnitude of these effects should be distinguished from their statistical detectability. With a corpus of this size, small differences are statistically distinguishable, but the register gap (0.749 versus 0.703) and the repetition gain (0.656–0.727) correspond to only small standardized effects (Cohen’s h ≈ 0.10 and ≈ 0.15, respectively). Likewise, the differences in held-out metrics between the interpretable models and the baselines, while their bootstrap confidence intervals exclude zero, are modest in absolute terms. The educational importance of effects of this size is not established by the present data and should be judged against the cost of the instructional changes they might motivate rather than assumed from statistical significance alone.

### 8.3 Pedagogical implications

Because these patterns are observational and correlational, the following implications are advanced as hypotheses for classroom research rather than as validated prescriptions. First, the negatively accelerated repetition curve in Figure 3 quantifies the marginal value of additional repetition: about two-thirds of the asymptotic gain was obtained in the first ten practices, which suggests the testable hypothesis that distributing repetitions across many lessons is more beneficial than clustering them within one (Ilari and Cho, 2023; Papageorgi et al., 2022). Second, the register effect in Figure 4 motivates the hypothesis that introducing low-register pitches before high-register pitches, with explicit respiratory and laryngeal preparation for the latter, would ease acquisition (Müllensiefen et al., 2022; Papageorgi et al., 2022). Third, the wide ability distribution in Figure 5 is consistent with a role for adaptive, learner-paced teaching in Chinese music classrooms, where content is often delivered uniformly to classes of forty or more students (Chung and Ho, 2022; Wei et al., 2022; Zhu et al., 2024). Each of these hypotheses would need to be tested in a controlled or longitudinal classroom study before being translated into curriculum policy.

These findings also have methodological implications for interpretable versus black-box student models in the musical context (Du and Butkaew, 2025; Liu et al., 2022; Wang et al., 2023; Zhang et al., 2024). Even though deep KT models tend to perform better on standard textual benchmarks (Zanellati et al., 2024), the current results show that interpretable models are able to capture significant structure relevant to classroom decisions and expose parameters that can directly inform teaching and curriculum design (Abdelrahman et al., 2023; Liu et al., 2022; Šarić-Grgić et al., 2024).

### 8.4 Limitations

Several limitations should be borne in mind. First, the singKT release provides no demographic information about the participants (age, sex, prior music education, socioeconomic status), all of which are known to affect singing acquisition (Luo and Guan, 2025; Pfordresher and Greenspon, 2025; Pfordresher, 2022). Second, the outcome is a single binary correctness flag emitted by an automated assessment engine; this is a coarse summary of the acoustic signal whose validity is not independently verified here, and it may mask systematic errors such as octave substitutions or vibrato artifacts, introducing measurement bias of unknown direction (Pfordresher and Greenspon, 2025). Third, attempts are not independent: they are nested within pitches, songs and learners, and the one-parameter IRT model further assumes a single latent ability dimension and a common discrimination across pitches. These assumptions are convenient but simplifying, and a multidimensional or multilevel treatment could alter the parameter estimates. Fourth, the learning curve is an across-learner average that is vulnerable to the survivorship effect noted in section 6, so it may overstate within-learner improvement. Fifth, learners self-selected onto the practice platform, so the sample may not represent the wider school population, limiting generalizability. Sixth, the data come from a specific instructional setting in Chinese schools, and caution is warranted in generalizing to other educational systems (Chung and Ho, 2022; Zhu et al., 2024). Seventh, the register analysis assumes that the dataset’s pitch identifiers are ordered by pitch height; the public singKT documentation does not explicitly confirm this mapping, so the register-compression result, although robust to the placement of the bin boundaries, rests on this labeling assumption and would benefit from an authoritative pitch-to-identifier key. Because the study is observational, none of its findings support causal or prescriptive conclusions.

## 9 Conclusion

This study characterized skill-acquisition trajectories in Chinese school-based music education, operationalized through note-level correctness, using the open singKT corpus. The analysis revealed a within-student repetition curve, a register effect on pitch difficulty and a wide range of latent learner ability, patterns that are consistent with theories of practice and singing development (Ericsson et al., 1993; Heathcote et al., 2000; Müllensiefen et al., 2022; Newell and Rosenbloom, 1981; Šarić-Grgić et al., 2024; Song et al., 2022). The IRT model outperformed the chance and per-pitch baselines in discriminative ability, and the BKT model attained the lowest cross-entropy and Brier scores and the highest accuracy on held-out attempts, with all comparisons accompanied by bootstrap confidence intervals; the absolute margins were nonetheless modest. Rather than prescribing practice, the findings are consistent with, and motivate controlled testing of, a curriculum model that provides low-register practice before high-register practice, distributes repetitions across lessons, and adapts pacing to a broad range of abilities in a typical classroom.

## 10 Future work

Four directions follow from this work. First, deep knowledge tracing models based on transformer attention or graph neural networks should be compared with the IRT and BKT models reported here, to determine whether the small accuracy gains they typically achieve justify their lower interpretability in classroom applications (Pandey and Karypis, 2019; Pu et al., 2022; Wang et al., 2025; Zanellati et al., 2024). Second, and equally important, the empirical trajectories should be connected to educational, developmental and instructional questions: how the identified trajectories inform theories of musical development, how they might optimize instructional sequencing, and how they could support curriculum design, ideally through controlled or longitudinal classroom studies rather than further modeling alone. Third, the trajectories should be related to demographic and motivational covariates gathered via complementary classroom surveys, to test mediation hypotheses about why some learners remain at the lower end of the ability distribution (Luo and Guan, 2025; Welch and Baxter, 2025). Fourth, the methodology should be extended to multimodal data, such as raw audio features, and the assessment engine should be co-modeled with the learner so that measurement error is represented explicitly (Liu et al., 2023; Pfordresher and Greenspon, 2025). Together these extensions would move the approach closer to the deployable, pedagogically grounded adaptive systems envisaged by recent reviews of music education technology (Huang et al., 2024; Wei et al., 2022).

## Data Availability

Publicly available datasets were analyzed in this study. This data can be found here: Repository name: GitHub (iTEC Lab, Huazhong University of Science and Technology). Direct data access link: https://github.com/itec-hust/singKT-dataset. No formal accession number is assigned to this open dataset hosted on GitHub.
